# Persons living with HIV with advanced HIV disease: need for novel care models

**DOI:** 10.1002/jia2.25210

**Published:** 2018-12-13

**Authors:** Chloe A Teasdale, Katharine Yuengling, Peter Preko, Maureen Syowai, Felix Ndagije, Miriam Rabkin, Elaine J Abrams, Wafaa M El‐Sadr

**Affiliations:** ^1^ ICAP‐Columbia University Mailman School of Public Health Columbia University New York NY USA; ^2^ Department of Epidemiology Mailman School of Public Health Columbia University New York NY USA; ^3^ College of Physicians and Surgeons Columbia University New York NY USA

**Keywords:** CD4 cell count, late ART initiation, loss to follow‐up, mortality, attrition, differentiated service delivery models

## Abstract

**Introduction:**

Despite increasing focus on test and treat strategies for people living with HIV (PLHIV), many continue to enrol late in care and initiate antiretroviral therapy (ART) when they have advanced HIV disease.

**Methods:**

We analyzed PLHIV ≥15 years of age starting ART in Ethiopia, Kenya, Mozambique and Tanzania from 2005 to 2015 based on CD4+ groups at ART initiation (≥200, 100 to 199, 50 to 99 and <50 cells/mm^3^) to examine attrition (loss to follow‐up (LTF) and death) using Kaplan‐Meier estimators and Cox proportional hazards models. LTF was defined as no clinic visit >6 months; deaths were ascertained from medical records.

**Results and discussion:**

A total of 305,443 PLHIV were included in the analysis: 118,580 (38.8%) CD4+ ≥200, 91,788 (30.1%) CD4+ 100 to 199, 44,029 (14.4%) CD4+ 50 to 99 and 51,046 (16.7%) CD4+ <50 cells/mm^3^. At 12 months after ART initiation, attrition for those with CD4+ ≥200, 100 to 199, 50 to 99 and <50 cells/mm^3^ was 21.3% (95% CI 21.1 to 21.6), 21.8% (95% CI 21.6 to 22.1), 27.3% (95% CI 26.9 to 27.7) and 33.6% (95% CI 33.2 to 34.0) respectively. In multivariable models, compared to PLHIV with CD4+ ≥200 cells/mm^3^, those with CD4+ 50 to 99 cells/mm^3^ had 29% increased risk of attrition (adjusted hazard ratio (AHR) 1.29, 95% CI 1.27 to 1.32) and those with <50 cells/mm^3^ had 56% increased risk of attrition (AHR 1.56, 95% CI 1.53 to 1.58). Men had higher attrition compared to women across all CD4+ groups and overall were 28% more likely to experience attrition (AHR 1.28, 95% CI 1.26 to 1.29). Even after ART initiation, PLHIV with advanced disease had notably inferior outcomes with substantial gradient within the low CD4+ strata highlighting the need for targeted interventions for these populations.

**Conclusions:**

Greater efforts, including the identification of effective differentiated service delivery models, are needed to ensure that all PLHIV starting treatment can garner the benefits from ART and achieve favourable outcomes.

## 1 Introduction

In 2015, World Health Organization (WHO) guidelines recommended treatment for all persons living with HIV (PLHIV) 1 in order to maximize the benefits of treatment for individuals with HIV and to prevent transmission to others 2, 3, 4. By the end of 2017, 80% of all low‐ and middle‐income countries and 94% of “fast track” countries (those accounting for 90% of new HIV infections globally), had adopted “treat all” strategies 5. However, many PLHIV, particularly those in resource limited settings (RLS), continue to enrol in HIV care and initiate treatment when they have advanced HIV disease, 6, 7 defined by WHO as CD4+ cell count of <200 cells/mm^3^. Initiation of antiretroviral therapy (ART) at advanced HIV disease is associated with poor treatment outcomes, including high rates of mortality and loss to follow‐up (LTF) 8, 9, 10. For this reason, in 2017, WHO issued guidelines for the management of patients with advanced HIV disease, including rapid ART initiation within seven days and same‐day initiation for those with no contraindications for treatment initiation 11.

Although previous analyses have shown that patients with CD4+ ≤200 cells/mm^3^, have higher risk of LTF and mortality, there have been few examinations of patient outcomes for individuals *within* this group, which represents up to half of all patients enrolling in care in some countries 7. Thus, there is a need to understand which patients with CD4+ ≤200 cells/mm^3^ are at most risk of poor outcomes. This information could be used to inform programmes and interventions, including developing differentiated service delivery (DSD) models, shaped to meet the needs of those most at risk. We report findings from an analysis of routinely collected data from four countries in sub‐Saharan Africa to measure outcomes among patients based on their immunologic status at the time of ART initiation.

## 2 Methods

We conducted a retrospective analysis of de‐identified patient data from health facilities in Ethiopia, Kenya, Mozambique and Tanzania. All health facilities received support from ICAP at Columbia University (ICAP) and offered a standard package of services, including HIV testing, pre‐ART and ART care, including prevention and treatment for opportunistic infections, as per each country's national guidelines. ICAP received funding for this work from the President's Emergency Plan For AIDS Relief through the United States Centers for Disease Control and Prevention (CDC). For these analyses, only de‐identified routinely collected data were used and investigators had no access to identifiable patient information. Ethics and administrative approvals were obtained in each of the four countries as well as from the Columbia University Medical Center institutional review board and the Associate Director of Science Office at the CDC.

The study population included all adult patients ≥15 years of age who enrolled in care from 1 January 2005 through 31 December 2014 and started ART as of 31 December 2015. Patients who reported prior ART and those whose ART initiation date was <6 months prior to the date when data collection ended at their health facility were excluded. Medical record data collected during routine clinic visits were entered into on‐site electronic databases by trained data capturers (ICAP supported data quality efforts at facilities). CD4+ cell count (CD4+) and WHO stage at ART initiation included measures recorded up to three months prior and one month after the start of treatment. Loss to follow‐up (LTF) after ART initiation was defined as not having a clinic visit for >6 months. Data on deaths and transfers out of facilities were ascertained from facility records. Time to LTF or death was calculated from the date of ART initiation to the date of death (if available) or the last visit date. Patients were divided into groups based on CD4+ cell count at ART initiation: CD4+ ≥200, 100 to 199, 50 to 99 and <50 cells/mm^3^. Patients missing CD4+ cell count at ART initiation were excluded from the analyses.

Chi square tests were used to compare the characteristics associated with having CD4+ cell count at ART initiation among all patients who started treatment. Survival analyses using Kaplan‐Meier estimators were used to calculate LTF, death and a combined attrition endpoint of LTF or death. Cox proportional hazard models adjusted for age, sex, country, year of ART and intrasite clustering were generated to compare attrition rates between patients based on immunologic status at ART initiation. The attrition endpoint was selected for modelling as mortality is under ascertained and deaths may have been classified as LTF. Statistical analyses were performed using SAS 9.3 (SAS Institute Inc., Cary, NC, USA) and Stata 12 (StataCorp., College Station, TX, USA).

## 3 Results and discussion

A total of 884,049 PLHIV were enrolled in HIV care at 350 health facilities in the four countries between 2005 and 2014. Among all PLHIV enrolled in care, 476,807 (53.9%) ever started ART through 2015 and 335,269 (70.3%) had a CD4+ cell count at the time of ART initiation. Among all PLHIV who started ART, men were somewhat more likely to have CD4+ cell count at treatment initiation compared to women (72.8% vs. 69.0%, *p *<* *0.0001), as were patients 30 years and older compared to those 15 to 29 years (71.4% vs. 68.0%, *p *<* *0.0001) and PLHIV with WHO clinical Stage 1 or 2 compared to those with 3 or 4 (75.7% vs. 72.3%, *p *<* *0.001). For this analysis, 29,826 (8.9%) PLHIV were excluded because they started ART <6 months before the end of data collection at their health facility.

Among 305,443 PLHIV included in the analysis, 118,580 (38.8%) had a CD4+ ≥200 cells/mm^3^ at ART initiation (median of 285 cells/mm^3^, interquartile range: 237 to 349 cells/mm^3^) and the remaining 186,863 (61.2%) PLHIV had low CD4+ (<200 cells/mm^3^) at ART initiation; 91,788 (30.1%) had CD4+ 100 to 199, 44,029 (14.4%) had CD4+ 50 to 99 cells/mm^3^ and 51,046 (16.7%) had CD4+ <50 cells/mm^3^ (Table 1). Over time, the proportion of PLHIV starting ART with CD4 < 200 cells/mm^3^ decreased from 76.8% of adult PLHIV starting ART (with CD4+ cell count measured) in the period 2005 to 2006 to 38.2% in 2013 to 2015.

**Table 1:** Characteristics at ART initiation among adults with HIV who initiated treatment and had CD4+ cell count in Ethiopia, Kenya, Mozambique and Tanzania 2005 to 2015 (N = 305,443)

	All	CD4+ count at ART initiation			
		>200	100 to 199	50 to 99	<50
	N (%)	N (%)	N (%)	N (%)	N (%)
	305,443 (100.0)	118,580 (38.8)	91,788 (30.1)	44,029 (14.4)	51,046 (16.7)
Median CD4 count at ART (IQR)	164 (78 to 255)	285 (237 to 349)	151 (125 to 176)	75 (62 to 87)	20 (7 to 35)
Country					
Ethiopia (73 sites)	71,601 (23.4)	20,296 (17.1)	26,109 (28.4)	13,303 (30.2)	11,893 (23.3)
Kenya (125 sites)	91,980 (30.1)	39,151 (33.0)	24,108 (26.3)	11,701 (26.6)	17,020 (33.3)
Mozambique (67 sites)	93,541 (30.6)	40,256 (33.9)	27,550 (30)	12,424 (28.2)	13,311 (26.1)
Tanzania (85 sites)	48,321 (15.8)	18,877 (15.9)	14,021 (15.3)	6601 (15.0)	8822 (17.3)
Sex					
Female	195,062 (63.9)	82,223 (69.3)	58,267 (63.5)	25,819 (58.6)	28,753 (56.3)
Male	110,381 (36.1)	36,357 (30.7)	33,521 (36.5)	18,210 (41.4)	22,293 (43.7)
Age at enrolment in HIV care					
Median (IQR)	34.5 (28.0 to 42.0)	33.2 (27.0 to 41.6)	35.0 (28.4 to 42.2)	35.0 (29.0 to 42.0)	35.0 (29.0 to 41.7)
15 to 19	7650 (2.5)	4247 (3.6)	1694 (1.8)	707 (1.6)	1002 (2.0)
0 to 29	86,595 (28.4)	37,506 (31.6)	24,741 (27.0)	11,112 (25.2)	13,236 (25.9)
30 to 39	114,317 (37.4)	41,350 (34.9)	34,710 (37.8)	17,531 (39.8)	20,726 (40.6)
40 to 49	63,747 (20.9)	22,380 (18.9)	20,080 (21.9)	9907 (22.5)	11,380 (22.3)
50+	33,134 (10.8)	13,097 (11.0)	10,563 (11.5)	4772 (10.8)	4702 (9.2)
Point of entry for care					
VCT	109,535 (35.9)	41,878 (35.3)	34,409 (37.5)	15,797 (35.9)	17,451 (34.2)
PMTCT	18,401 (6.0)	11,563 (9.8)	4316 (4.7)	1364 (3.1)	1158 (2.3)
TB/HIV	8674 (2.8)	2818 (2.4)	2469 (2.7)	1431 (3.3)	1956 (3.8)
Inpatient	15,358 (5.0)	4765 (4.0)	4276 (4.7)	2596 (5.9)	3721 (7.3)
Outpatient	48,289 (15.8)	16,343 (13.8)	14,981 (16.3)	7849 (17.8)	9116 (17.9)
Other	80,566 (26.4)	31,826 (26.8)	23,920 (26.1)	11,312 (25.7)	13,508 (26.5)
Unknown	24,620 (8.1)	9387 (7.9)	7417 (8.1)	3680 (8.4)	4136 (8.1)
WHO stage at ART					
I	42,395 (17.3)	21,768 (23.0)	12,993 (17.5)	3877 (10.9)	3757 (9.1)
II	64,645 (26.3)	25,641 (27.1)	22,072 (29.7)	8569 (24.0)	8363 (20.4)
III	114,107 (46.5)	39,790 (42.1)	33,277 (44.8)	19,003 (53.2)	22,037 (53.6)
IV	24,432 (9.9)	7280 (7.7)	5933 (8.0)	4282 (12.0)	6937 (16.9)
Missing WHO stage at ART	59,864 (19.6)	24,101 (20.3)	17,513 (19.1)	8298 (18.8)	9952 (19.5)
Year of enrolment in HIV care					
2005 to 2006	44,334 (14.5)	12,956 (10.9)	15,165 (16.5)	7458 (16.9)	8755 (17.2)
2007 to 2008	85,214 (27.9)	27,542 (23.2)	28,640 (31.2)	13,704 (31.1)	15,328 (30.0)
2009 to 2010	83,188 (27.2)	33,152 (28.0)	24,863 (27.1)	11,586 (26.3)	13,587 (26.6)
2011 to 2012	61,322 (20.1)	27,741 (23.4)	16,252 (17.7)	7859 (17.8)	9470 (18.6)
2012 to 2014	31,385 (10.3)	17,189 (14.5)	6868 (7.5)	3422 (7.8)	3906 (7.7)
Year initiated ART					
2005 to 2006	33,122 (10.8)	7690 (6.5)	11,370 (12.4)	6386 (14.5)	7676 (15.0)
2007 to 2008	75,350 (24.7)	20,937 (17.7)	26,656 (29.0)	13,138 (29.8)	14,619 (28.6)
2009 to 2010	80,906 (26.5)	29,153 (24.6)	26,139 (28.5)	11,864 (26.9)	13,750 (26.9)
2011 to 2012	71,257 (23.3)	33,115 (27.9)	19,016 (20.7)	8696 (19.8)	10,430 (20.4)
2012 to 2014	43,614 (14.3)	26,721 (22.5)	8472 (9.2)	3898 (8.9)	4523 (8.9)
2015	1194 (0.4)	964 (0.8)	135 (0.1)	47 (0.1)	48 (0.1)

ART, antiretroviral therapy; IQR, interquartile range; WHO, World Health Organization.

**Figure 1:**
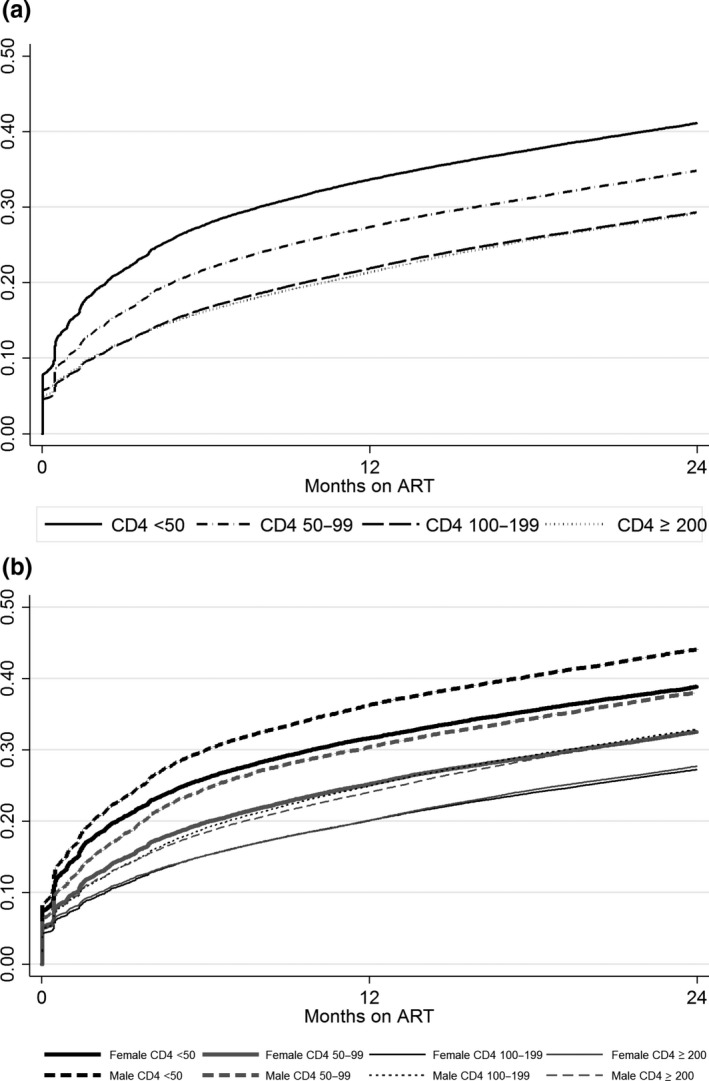
(**a**) Attrition (loss to follow‐up and death) among adults based on CD4 cell count at ART initiation in Ethiopia, Kenya, Mozambique and Tanzania 2005 to 2015 (N = 305,443). (**b**) Attrition among adults by sex and CD4+ cell count at ART initiation. ART, antiretroviral therapy.

At 12 months after ART initiation, 21.3% (95% CI 21.1 to 21.6) of PLHIV with CD4+ ≥200 cells/mm^3^ had been LTF or had died compared to 21.8% (95% CI 21.6 to 22.1) among those with CD4+ 100 to 199 cells/mm^3^, 27.3% (95% CI 26.9 to 27.7) among those with CD4+ 50 to 99 cells/mm^3^ and 33.6% (95% CI 33.2 to 34.0) among those with CD4+ <50 cells/mm^3^ (Table 2). In multivariable models, compared to PLHIV with CD4+ ≥200 cells/mm^3^, those with CD4+ 100 to 199 cells/mm^3^ had 9% increased risk of attrition (adjusted hazard ratio (AHR) 1.09, 95% CI 1.07 to 1.11), those with CD4+ 50 to 99 cells/mm^3^ had 29% increased risk of attrition (AHR 1.29, 95% CI 1.27 to 1.32) and PLHIV with CD4+ <50 cells/mm^3^ had 56% increased risk (AHR 1.56, 95% CI 1.53 to 1.58) (Figure 1a, Table 3). Women had significantly lower attrition compared to men at all time points and across all CD4+ cell groups (log rank *p *<* *0.0001) (Figure 1b); overall men were 28% more likely to experience attrition compared to women in multivariable models (AHR 1.28, 95% CI 1.26 to 1.29) (Table 3). Attrition also significantly increased over time in adjusted models with patient enrolled in later years more likely to experience attrition (Table 3).

**Table 2:** Estimates of LTF, death and combined attrition (LTF and death) among adult PLHIV initiating antiretroviral therapy in Ethiopia, Kenya, Mozambique and Tanzania 2004 to 2015 (N = 305,443)

	All				CD4 < 50				CD4 50 to 99				CD4 100 to 199				CD4 ≥ 200			
	Six months		Twelve months		Six months		Twelve months		Six months		Twelve months		Six months		Twelve months		Six months		Twelve months	
	Est.	95% CI	Est.	95% CI	Est.	95% CI	Est.	95% CI	Est.	95% CI	Est.	95% CI	Est.	95% CI	Est.	95% CI	Est.	95% CI	Est.	95% CI
Loss to follow‐up																				
Overall	15.4	15.2 to 15.5	20.3	20.1 to 20.4	20.1	19.7 to 20.5	25.4	25.0 to 25.8	16.7	16.4 to 17.1	21.6	21.2 to 22.0	13.5	13.3 to 13.7	18.3	18.0 to 18.5	14.4	14.2 to 14.6	19.2	19.0 to 19.4
Female	14.4	14.3 to 14.6	19.1	18.9 to 19.3	19.2	18.8 to 19.7	24.3	23.8 to 24.9	15.5	15.1 to 16.0	20.2	19.7 to 20.7	12.6	12.3 to 12.9	17.0	16.7 to 17.4	13.8	13.6 to 14.1	18.5	18.2 to 18.7
Male	17.1	16.8 to 17.3	22.4	22.1 to 22.6	21.2	20.7 to 21.8	26.8	26.2 to 27.4	18.5	17.9 to 19.1	23.6	23.0 to 24.3	15.1	14.7 to 15.5	20.5	20.0 to 20.9	15.8	15.4 to 16.1	20.9	20.5 to 21.4
Country																				
Ethiopia	12.3	12.0 to 12.5	17.6	17.3 to 17.9	18.6	17.9 to 19.4	25.1	24.2 to 25.9	14.7	14.1 to 15.3	20.3	19.6 to 21.0	10.6	10.2 to 11.0	15.7	15.2 to 16.1	9.3	8.9 to 9.8	14.3	13.8 to 14.8
Kenya	14.4	14.2 to 14.7	18.7	18.4 to 18.9	18.3	17.7 to 18.9	23.1	22.4 to 23.8	16.1	15.4 to 16.8	20.0	19.2 to 20.7	13.6	13.2 to 14.0	17.8	17.3 to 18.3	12.8	12.5 to 13.2	17.0	16.6 to 17.4
Mozambique	19.9	19.7 to 20.2	25.6	25.3 to 25.9	24.7	24.0 to 25.5	30.3	29.5 to 31.1	20.7	20.0 to 21.5	26.2	25.4 to 27.0	17.0	16.6 to 17.5	22.5	22.0 to 23.1	20.1	19.7 to 20.5	26.1	25.7 to 26.6
Tanzania	12.9	12.6 to 13.2	16.7	16.3 to 17.0	18.5	17.7 to 19.4	22.6	21.7 to 23.6	14.4	13.5 to 15.3	18.3	17.3 to 19.3	11.7	11.1 to 12.3	15.5	14.9 to 16.2	10.8	10.4 to 11.3	14.4	13.9 to 14.9
Age at enrolment (years)																				
15 to 19	23.7	22.7 to 24.7	30.3	29.2 to 31.4	21.5	19.0 to 24.3	27.7	24.9 to 30.8	20.8	17.9 to 24.1	26.7	23.5 to 30.3	20.0	18.1 to 22.0	26.8	24.7 to 29.1	26.2	24.8 to 27.5	32.9	31.4 to 34.4
20 to 29	18.1	17.8 to 18.3	23.9	23.6 to 24.2	21.9	21.2 to 22.7	28.3	27.5 to 29.2	18.5	17.8 to 19.3	24.2	23.4 to 25.1	16.1	15.7 to 16.6	21.9	21.3 to 22.4	17.9	17.5 to 18.3	23.6	23.2 to 24.1
30 to 39	14.5	14.3 to 14.7	19.3	19.1 to 19.6	19.7	19.1 to 20.3	24.9	24.3 to 25.6	16.6	16.0 to 17.1	21.4	20.8 to 22.1	12.7	12.4 to 13.1	17.4	17.0 to 17.8	12.8	12.5 to 13.1	17.5	17.1 to 17.9
40 to 49	13.3	13.0 to 13.6	17.3	17.0 to 17.6	19.0	18.3 to 19.8	23.4	22.6 to 24.3	15.6	14.9 to 16.4	19.8	19.0 to 20.6	11.3	10.9 to 11.8	15.4	14.8 to 15.9	11.2	10.8 to 11.7	15.1	14.6 to 15.6
50+	13.4	13.0 to 13.8	17.4	16.9 to 17.8	19.0	17.9 to 20.2	23.4	22.2 to 24.8	14.8	13.8 to 15.9	19.2	18.1 to 20.4	13.1	12.4 to 13.7	17.1	16.4 to 17.9	11.3	10.8 to 11.9	14.8	14.2 to 15.5
Death																				
Overall	4.3	4.2 to 4.4	5.1	5.0 to 5.2	9.4	9.1 to 9.7	10.9	10.6 to 11.2	6.0	5.8 to 6.3	7.2	7.0 to 7.5	3.6	3.4 to 3.7	4.3	4.2 to 4.5	2.1	2.0 to 2.2	2.6	2.5 to 2.7
Female	3.4	3.4 to 3.5	4.1	4.0 to 4.2	8.3	7.9 to 8.6	9.5	9.1 to 9.9	5.1	4.8 to 5.4	6.2	5.9 to 6.5	3.0	2.8 to 3.1	3.6	3.5 to 3.8	1.6	1.5 to 1.7	2.0	1.9 to 2.1
Male	5.8	5.6 to 5.9	6.9	6.7 to 7.1	10.9	10.5 to 11.3	12.7	12.2 to 13.2	7.3	6.9 to 7.7	8.7	8.3 to 9.2	4.6	4.3 to 4.8	5.5	5.3 to 5.8	3.1	2.9 to 3.3	3.9	3.7 to 4.1
Country																				
Ethiopia	4.4	4.2 to 4.5	5.3	5.1 to 5.5	9.6	9.1 to 10.2	11.4	10.8 to 12.1	6.1	5.7 to 6.5	7.3	6.8 to 7.8	3.3	3.1 to 3.6	4.0	3.8 to 4.3	1.7	1.5 to 1.9	2.2	2.0 to 2.4
Kenya	2.5	2.4 to 2.6	3.1	3.0 to 3.2	5.8	5.5 to 6.2	7.0	6.6 to 7.4	3.9	3.5 to 4.3	4.8	4.4 to 5.3	2.3	2.1 to 2.5	2.8	2.6 to 3.0	0.9	0.8 to 1.0	1.2	1.1 to 1.3
Mozambique	4.3	4.2 to 4.4	5.2	5.1 to 5.4	9.5	9.0 to 10.1	11.1	10.5 to 11.7	6.1	5.7 to 6.6	7.4	6.9 to 7.9	3.7	3.5 to 3.9	4.6	4.3 to 4.8	2.5	2.3 to 2.6	3.2	3.0 to 3.4
Tanzania	7.3	7.1 to 7.6	8.5	8.2 to 8.7	15.5	14.7 to 16.3	17.2	16.3 to 18.1	9.3	8.6 to 10.1	10.7	10.0 to 11.6	5.9	5.5 to 6.3	7.0	6.6 to 7.5	4.1	3.8 to 4.4	4.8	4.5 to 5.2
Age at enrolment (years)																				
15 to 19	3.5	3.0 to 3.9	4.3	3.8 to 4.8	8.5	6.8 to 10.6	9.9	8.0 to 12.2	5.7	4.1 to 8.0	7.1	5.2 to 9.6	3.6	2.8 to 4.7	4.2	3.3 to 5.4	1.8	1.4 to 2.3	2.4	1.9 to 3.0
20 to 29	3.7	3.6 to 3.8	4.4	4.3 to 4.6	8.5	8.0 to 9.0	10.0	9.5 to 10.6	5.4	5.0 to 5.9	6.6	6.1 to 7.1	3.3	3.0 to 3.5	3.9	3.7 to 4.2	1.9	1.7 to 2.0	2.2	2.1 to 2.4
30 to 39	4.4	4.2 to 4.5	5.2	5.0 to 5.3	9.4	9.0 to 9.9	11.0	10.5 to 11.5	6.0	5.6 to 6.4	7.1	6.7 to 7.5	3.5	3.3 to 3.8	4.2	4.0 to 4.44	1.9	1.8 to 2.1	2.4	2.3 to 2.6
40 to 49	4.6	4.4 to 4.8	5.6	5.4 to 5.7	9.6	9.1 to 10.2	11.0	10.4 to 11.7	6.4	5.9 to 6.9	7.8	7.3 to 8.4	3.7	3.4 to 3.9	4.5	4.2 to 4.8	2.2	2.0 to 2.4	2.9	2.7 to 3.1
50+	5.0	4.8 to 5.3	6.1	5.8 to 6.3	11.5	10.6 to 12.5	12.8	11.8 to 13.9	6.7	6.0 to 7.5	7.9	7.2 to 8.8	4.1	3.7 to 4.5	5.3	4.9 to 5.8	3.0	2.7 to 3.4	3.7	3.4 to 4.1
Attrition																				
Overall	19.0	18.9 to 19.2	24.4	24.2 to 24.5	27.7	27.3 to 28.1	33.6	33.2 to 34.0	21.8	21.4 to 22.2	27.3	26.9 to 27.7	16.6	16.4 to 16.8	21.8	21.6 to 22.1	16.2	16.0 to 16.4	21.3	21.1 to 21.6
Female	17.4	17.2 to 17.6	22.5	22.3 to 22.7	26.0	25.5 to 26.5	31.6	31.1 to 32.2	19.9	19.4 to 20.4	25.2	24.6 to 25.7	15.2	14.9 to 15.5	20.1	19.8 to 20.4	15.2	15.0 to 15.5	20.1	19.8 to 20.4
Male	21.9	21.7 to 22.2	27.8	27.5 to 28.1	30.0	29.3 to 30.6	36.2	35.6 to 36.9	24.5	23.9 to 25.2	30.4	29.7 to 31.1	19.0	18.6 to 19.5	24.9	24.5 to 25.4	18.4	18.0 to 18.8	24.0	23.6 to 24.5
Country																				
Ethiopia	16.1	15.8 to 16.4	22.0	21.6 to 22.3	26.5	25.7 to 27.3	33.7	32.8 to 34.5	19.9	19.2 to 20.6	26.1	25.4 to 26.9	13.6	13.2 to 14.0	19.1	18.6 to 19.6	10.9	10.4 to 11.3	16.2	15.7 to 16.7
Kenya	16.6	16.4 to 16.9	21.2	20.9 to 21.5	23.2	22.6 to 23.9	28.6	27.9 to 29.3	19.4	18.7 to 20.1	23.9	23.1 to 24.7	15.6	15.1 to 16.1	20.1	19.6 to 20.6	13.7	13.3 to 14.0	18.0	17.6 to 18.3
Mozambique	23.4	23.1 to 23.7	29.6	29.3 to 29.9	32.0	31.3 to 32.9	38.2	37.3 to 39.0	25.6	24.9 to 26.4	31.7	30.9 to 32.6	20.1	19.6 to 20.6	26.1	25.6 to 26.6	22.1	21.7 to 22.5	28.5	28.0 to 28.9
Tanzania	19.3	19.0 to 19.7	23.8	23.4 to 24.2	31.4	30.4 to 32.4	36.1	35.1 to 37.2	22.4	21.4 to 23.5	27.2	26.1 to 28.3	16.9	16.3 to 17.5	21.5	20.8 to 22.2	14.5	14.0 to 15.0	18.6	18.0 to 19.2
Age at enrolment (years)																				
15 to 19	26.4	25.4 to 27.4	33.3	32.2 to 34.4	28.3	25.5 to 31.2	35.0	32.0 to 38.1	25.4	22.3 to 28.8	31.9	28.5 to 35.7	22.9	20.9 to 25.0	30.0	27.8 to 32.3	27.5	26.2 to 28.9	34.5	33.1 to 36.1
20 to 29	21.1	20.8 to 21.4	27.3	27.0 to 27.6	28.7	27.9 to 29.5	35.6	34.8 to 36.5	23.0	22.2 to 23.8	29.2	28.3 to 30.1	18.9	18.4 to 19.4	25.0	24.4 to 25.5	19.4	19.0 to 19.8	25.3	24.9 to 25.8
30 to 39	18.3	18.1 to 18.5	23.6	23.3 to 23.8	27.4	26.7 to 28.0	33.3	32.6 to 33.9	21.6	21.0 to 22.2	27.1	26.4 to 27.8	15.8	15.4 to 16.2	20.9	20.5 to 21.3	14.5	14.1 to 14.8	19.5	19.1 to 19.9
40 to 49	17.3	17.0 to 17.6	22.0	21.6 to 22.3	27.0	26.2 to 27.8	32.0	31.1 to 32.9	21.1	20.3 to 21.9	26.1	25.2 to 27.0	14.6	14.1 to 15.1	19.2	18.6 to 19.7	13.2	12.8 to 13.7	17.6	17.1 to 18.1
50+	17.8	17.4 to 18.2	22.4	22.0 to 22.9	28.5	27.2 to 29.8	33.4	32.0 to 34.8	20.6	19.5 to 21.8	25.7	24.4 to 27.0	16.6	15.9 to 17.4	21.6	20.8 to 22.4	14.0	13.4 to 14.6	18.0	17.4 to 18.7

LTF, loss to follow‐up; PLHIV, people living with HIV.

**Table 3:** Multivariable model of the relationship between CD4 cell count at ART initiation and combine attrition (loss to follow‐up and death) among adults in Ethiopia, Kenya, Mozambique and Tanzania 2005 to 2015 (N = 305,443)

	Univariable			Multivariable		
	HR	95% CI	*p*‐value	aHR	95% CI	*p*‐value
CD4 group at ART						
<50	1.50	1.48 to 1.52	<0.0001	1.56	1.53 to 1.58	<0.0001
50 to 99	1.22	1.20 to 1.24	<0.0001	1.29	1.27 to 1.32	<0.0001
100 to 199	1.03	1.01 to 1.04	0.0002	1.09	1.07 to 1.11	<0.0001
≥200	1	ref		1	ref	
Age at enrolment						
15 to 19	1.57	1.52 to 1.63	<0.0001	1.62	1.56 to 1.69	<0.0001
20 to 29	1.22	1.19 to 1.24	<0.0001	1.29	1.27 to 1.32	<0.0001
30 to 39	1.05	1.03 to 1.07	<0.0001	1.08	1.06 to 1.10	<0.0001
40 to 49	0.97	0.95 to 0.99	0.0081	0.97	0.95 to 0.99	0.0055
50+	1	ref		1	ref	
Gender						
Female	1	ref		1	ref	
Male	1.23	1.19 to 1.27	<0.0001	1.28	1.26 to 1.29	<0.0001
Country						
Tanzania	1	ref		1	ref	
Mozambique	1.35	1.32 to 1.37	<0.0001	1.33	1.31 to 1.36	<0.0001
Kenya	0.99	0.98 to 1.01	0.55	1.00	0.98 to 1.01	0.58
Ethiopia	0.84	0.82 to 0.86	<0.0001	0.81	0.79 to 0.83	<0.0001
Year of enrolment						
2005 to 2006	1	ref		1	ref	
2007 to 2008	1.10	1.08 to 1.12	<0.0001	1.11	1.09 to 1.13	<0.0001
2009 to 2010	1.12	1.10 to 1.14	<0.0001	1.16	1.14 to 1.18	<0.0001
2011 to 2012	1.31	1.29 to 1.34	<0.0001	1.32	1.30 to 1.35	<0.0001
2013 to 2014	1.41	1.37 to 1.45	<0.0001	1.38	1.35 to 1.42	<0.0001

AHR, adjusted hazard ratio; ART, antiretroviral therapy.

In this large cohort of PLHIV enrolled in HIV care across four countries between 2005 and 2014, more than half (53.9%) started ART and among those with CD4+ cell count at treatment initiation, 60% had advanced HIV disease. Within the group of patients with low CD4+ cell count (<200 cells/mm^3^), we observed significant differences in the combined attrition endpoint of LTF and mortality. Patients with CD4+ cell count ≥200 cells/mm^3^ and those with CD4 100 to 199 cells/mm^3^ had similar outcomes, with roughly 20% experiencing LTF or death by 12 months after ART initiation, whereas among those with CD4+ cell count of 50 to 100 and CD4+ <50 cells/mm^3^, attrition ranged from 27% to 34%. PLHIV with the most advanced disease (CD4+ <50 cells/mm^3^) had a 56% increased risk of LTF or death compared to those with CD4+ ≥200 cells/mm^3^. Men were also more likely to experience attrition compared to women regardless of immunologic status.

While the proportion of PLHIV starting ART with advanced disease has declined in recent years, they remain a significant portion of all patients enrolling in care and starting treatment in reports from across sub Saharan Africa 6, 7, 12. A retrospective analysis of almost 700,00 adults across 10 countries also showed a decline in advanced disease status at ART initiation but in several countries, up to 20% of patients continue to start treatment with CD4 < 100 copies/mm^3^.^13^ Recent data from the Population‐based HIV Impact Assessments in Malawi, Zambia and Zimbabwe indicate that among previously undiagnosed adults identified as HIV‐positive through these surveys, 45% to 50% had CD4+ <350 cells/mm^3^, with men being significantly more likely to have CD4+ cell count below this threshold compared to women 14. These data highlight the ongoing challenge that many countries face in identifying all those with HIV infection and the magnitude of the continuing problem of late enrolment in care and advanced disease at initiation of ART.

Our findings confirm the existing evidence of poorer outcomes among patients initiating ART at lower CD4+ cell count 8, 9. However, we expand on those findings with our analyses and demonstrate a significant gradient of risk among those with CD4+ <200 cells/mm^3^ at ART initiation, with the highest risk of poor outcomes found in those in the lowest CD4+ cell count group (<50 cells/mm^3^). A previous analysis from South Africa examining CD4 at ART on risk of LTF found no association between lower CD4 at treatment initiation and LTF for patients with CD4 < 300 when unascertained deaths were accounted for 15. Our analysis utilized a combined endpoint of attrition including LTF and death which may account for the difference in our findings. Furthermore, our data also show that outcomes were quite similar for patients with CD4+ cell count of 100 to 199 and those with CD4 ≥ 200 cells/mm^3^ at ART. This finding is interesting as CD4+ <200 continues to be used as the “cutoff” for defining advanced disease, but our data suggest that there is heterogeneity in outcomes within that group and that that a CD4+ cell count of <100 cells/mm^3^ may be a more sensitive cutoff for identifying PLHIV who are at high risk for poor outcomes.

We also observed significantly inferior outcomes for men compared to women regardless of immunologic status, with men experiencing higher rates of attrition within all CD4+ cell count groups. This is a novel finding as most previous findings of higher attrition among men have assumed that their worse outcomes are due to initiation of ART at more advanced stages of HIV disease 16, 17. Our findings show that compared to women within CD4 cell count strata at ART initiation, men have poorer outcomes. Further research is needed to understand why men are more likely to be lost after ART initiation but some reasons may include higher rates of alcohol use and higher perceived stigma which may contribute to worse outcomes 18, 19, 20. We also found increasing overall attrition over time which is in keeping with other analyses 21, 22, 23. This finding could reflect decreasing focus on retention as programmes scale‐up services but could also be driven, in part, by increasing availability of ART services and higher rates of undocumented transfers between health facilities.

A wide range of differentiated ART service models (DARTS) have been developed for stable patients on ART, but DARTS models for patients with advanced disease and for men are less common 24, 25, 26. The WHO 2017 guidelines on managing patients with advanced HIV call for rapid initiation of treatment, prophylaxis and pre‐emptive treatment for TB and other opportunistic infections and adapted adherence support 11 which may include more intensive follow‐up schedules and home‐based care 27. There are limited data on effective DARTS models specifically targeted to men but these could include workplace‐based services, evening and weekend hours, men‐only community adherence groups, and inclusion of income‐generating activities.

Finally, our findings underscore the importance of routine CD4+ cell count as a tool for identifying patients at high risk for poor outcomes (although we do not advocate delaying ART initiation while awaiting CD4 results). While scaling up access to viral load monitoring is a priority, CD4+ cell count testing at treatment initiation remains important in order to identify patients who are at greatest risk for poor outcomes and to guide tailored services.

The study has several strengths. The data used for the analysis we present are derived from a large cohort of PLHIV in RLS collected during routine care offered at publicly supported facilities across four countries. The dataset also includes a long period of follow‐up allowing us to examine changes over time. Limitation of the analysis include missing CD4+ cell counts for almost 30% of the patients who started treatment, who were slightly more likely to be female, younger and to have more advanced disease. While the differences observed in the characteristics of PLHIV with and without CD4+ were small (less than 4%), they were statistically significant as a result of the very large sample size of almost half a million patients and it is unlikely that the missing data altered the overall findings. An additional limitation is the potential for silent or undocumented transfer of patients between facilities which could have led to an overestimate of LTF and combined attrition in our analysis. Previous studies have estimated that roughly 20% of patients who appear to have disengaged from care are alive and receiving treatment at a different health facility 28, 29, 30, 31, 32 and these findings should be considered in relation to our estimates.

## 4 Conclusions

Overall, our findings show that a significant proportion of PLHIV in RLS continue to initiate ART with advanced HIV disease which contributes to poor outcomes, particularly for those with very low CD4+ cell counts and among men across all CD4+ cell count strata. Greater efforts, including the identification of DSD models, are needed to ensure that all PLHIV starting treatment can garner the benefits from ART and achieve favourable outcomes.

## Competing interests

None of the authors have competing interests.

## Authors’ contributions

WME, EJA and CAT conceptualized the analysis. CAT, KY, WME and EJA developed the analysis plan. CAT and KY conducted the data analysis. All authors contributed to manuscript writing and review.
